# Dramatic activation of an antibody by a single amino acid change in framework

**DOI:** 10.1038/s41598-021-01530-w

**Published:** 2021-11-16

**Authors:** Wei-Ching Liang, Jianping Yin, Patrick Lupardus, Jianhuan Zhang, Kelly M. Loyet, Jawahar Sudhamsu, Yan Wu

**Affiliations:** 1 Department of Antibody Engineering, Genentech Inc., 1 DNA Way, South San Francisco, CA 94080 USA; 2 Department of Structural Biology, Genentech, Inc., 1 DNA Way, South San Francisco, CA 94080 USA; 3 Department of Biochemical and Cellular Pharmacology, Genentech, 1 DNA Way, South San Francisco, CA 94080 USA; 4 Department of Discovery Oncology, Genentech, Inc., 1 DNA Way, South San Francisco, CA 94080 USA

**Keywords:** Drug discovery, Molecular biology, Structural biology

## Abstract

Antibody function is typically entirely dictated by the Complementarity Determining Regions (CDRs) that directly bind to the antigen, while the framework region acts as a scaffold for the CDRs and maintains overall structure of the variable domain. We recently reported that the rabbit monoclonal antibody 4A11 (rbt4A11) disrupts signaling through both TGFβ2 and TGFβ3 (Sun et al. in Sci Transl Med, 2021. https://doi.org/10.1126/scitranslmed.abe0407). Here, we report a dramatic, unexpected discovery during the humanization of rbt4A11 where, two variants of humanized 4A11 (h4A11), v2 and v7 had identical CDRs, maintained high affinity binding to TGFβ2/3, yet exhibited distinct differences in activity. While h4A11.v7 completely inhibited TGFβ2/3 signaling like rbt4A11, h4A11.v2 did not. We solved crystal structures of TGFβ2 complexed with Fab fragments of h4A11.v2 or h4A11.v7 and identified a novel interaction between the two heavy chain molecules in the 2:2 TGFb2:h4A11.v2-Fab complex. Further characterization revealed that framework residue variations at either position 19, 79 or 81 (Kabat numbering) of the heavy chain strikingly converts h4A11.v2 into an inhibitory antibody. Our work suggests that in addition to CDRs, framework residues and interactions between Fabs in an antibody could be engineered to further modulate activity of antibodies.

## Introduction

Transforming Growth Factor-beta (TGFβ) is a key driver of fibrogenesis. There are three TGFβ isoforms with highly homologous receptor-binding domains with high sequence similarity, and have similar effects on target cells in vitro. Unlike TGFβ1, both TGFβ2 and TGFβ3 are expressed at elevated levels in human lung and liver fibrotic tissues and can be activated via distinct mechanisms^1^. Further, inhibiting both TGFβ2 and TGFβ3 while sparing TGFβ1 could alleviate lung fibrosis while avoiding toxicity concerns associated with pan-TGFβ blockade. We recently identified an isoform-selective rabbit monoclonal antibody (rbt4A11) that binds mature forms of both TGFβ2 and TGFβ3 with picomolar (pM) affinity, but shows no binding to the mature form of TGFβ1. It also has potent inhibitory activity against both TGFβ2 and TGFβ3 with the potential to be developed as a therapeutic for lung fibrosis. Humanization of rbt4A11 was performed to enable development of a potential clinical candidate for non-human primate pharmacokinetics and safety studies. Unlike previously described pan-TGFβ blocking antibodies such as Fresolimumab (Sanofi), the structure of a humanized rbt4A11 Fab (h4A11.v7) complexed with TGFβ2 shows that the epitope for h4A11.v7 is not near the TGFβR1/R2 binding site, and it does not sterically compete for receptor binding. Instead h4A11.v7 Fabs induce a conformational change in TGFβ2, causing the two monomers to “pinch” together by several degrees, demonstrating an allosteric mechanism of inhibition^1^.

For humanization of rbt4A11, we adopted well-accepted approaches in humanization of rodent (mouse and rat) monoclonal antibodies (mAbs) by grafting complementarity-determining regions (CDRs) and framework residues at the Vernier zone of rabbit antibody onto the human immunoglobulin germline gene segments which shared the highest sequence identity^2,3^. Combining this approach with the framework shuffling strategy, an efficient and effective way to manipulate and improve antibody properties in humanization of murine mAbs allowed us to rapidly select the most favorable combinations of human germline frameworks to maintain the affinity and activity of the parental antibody^4,5^.

In the course of rbt4A11 humanization, the entire panel of humanized 4A11 (h4A11) variants exhibited pM binding affinities very similar to the parental clone, but surprisingly varied in inhibition properties for TGFβ2 and TGFβ3 in a cell-based activity assay. To better understand the molecular mechanisms that conferred drastically different functionality of the different humanized variants that still had almost identical CDRs, we solved crystal structures of antigen-binding fragments (Fabs) from a complete blocker (h4A11.v7)(previous work)^1^ and an incomplete blocker (h4A11.v2)(current work) in complex with human TGFβ2. The structures revealed specific interactions between framework regions in the heavy chains of the two h4A11.v2 Fabs bound to the TGFβ2 dimer. Disruption of this interaction by a single amino acid variation at either position 19, 79 or 81 in the heavy chain framework converts h4A11.v2 to a ‘complete blocker’, similar to h4A11.v7 and rbt4A11.

## Results

### Humanization of rabbit mAb 4A11 by grafting CDRs to the closest human frameworks

**Figure 1:**
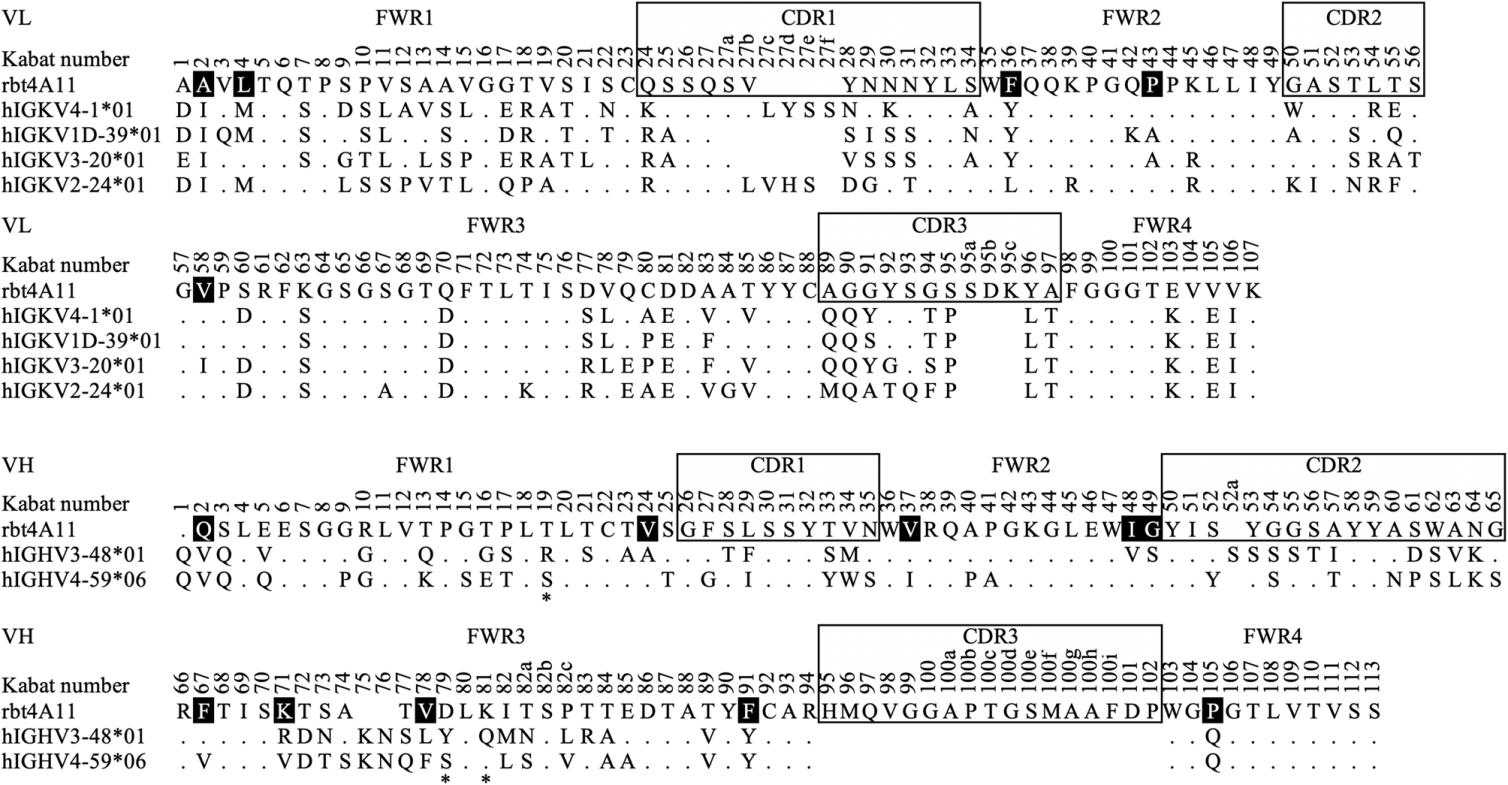
Amino acid sequence alignment of VL and VH of rbt4A11 with the closest human germline acceptor frameworks for humanization. The variable light chain (VL) and heavy chain (VH) domain of rbt4A11 was aligned with its closets human germlines to specify the different (letter) and identical (dot) amino acid residues in framework regions (FWR) and complementarity-determining regions (CDRs) by Kabat numbering scheme^6^. Six CDRs (box) and fifteen framework residues at Vernier zone (black fill) of rbt4A11 were fused in-frame to individual human germline acceptor framework for shuffling. Three positions (19, 79, 81) at the heavy chain framework guided by structure complex analysis were residues used to modulate antibody function (asterisk). The positions with sequence deletion and undefined heavy chain germline CDR3 were shown in blanks.

Rabbit monoclonal antibody 4A11 (rbt4A11) was humanized in a two-step strategy. First, the human germline frameworks that are closest to the variable light chain (VL) and variable heavy chain (VH) of rbt4A11 were identified based on the selection criteria of the highest amino acid sequence identity in variable region, frequent usage in antibody repertoire, and functional genes in different germline family. In total, we found four human light chain germline genes (IGKV1D-39*01, IGKV4-1*01, IGKV3-20*01, IGKV2-24*01) and two human heavy chain germline genes (IGHV3-48*01, IGHV4-59*06) as the closest acceptor frameworks, which were 62%, 61%, 54% and 54% identical to the VL, and 57% and 52% identical to the VH of rbt4A11, respectively. Subsequently, the rbt4A11 CDRs, which covered the definition of Kabat and Chothia^6^, and fifteen rabbit framework residues at the Vernier zone (position 2, 4, 36, 43 and 58 of VL, and position 2, 24, 37, 48, 49, 67, 71, 78, 91 and 105 of VH) were grafted onto each acceptor framework to generate four humanized VL and two humanized VH gene segments for molecular cloning (Fig. 1). To assess the best humanized variants, a panel of eight humanized 4A11 variants (h4A11.v1–h4A11.v8) was generated through mixing and matching the cloned humanized gene segments and expressing each variant as human IgG1 for further characterization (Table 1). The expression and purification profiles of all humanized variants were good (> 99% monomer) with no noticeable differences between germline usage. The variable domains of these eight humanized 4A11 variants comprised an identity of 77–81% in the human immunoglobulin germline sequence, which were further improved by framework Vernier permutation from original rabbit residues to the corresponding human germline residues to meet World Health Organization standards as a humanized antibody (data not shown).

**Table 1:** Functional characterization of primary humanized 4A11 variants.

Variants	Human germline acceptor framework		TGFβ binding by Biacore SPR (K_D_: pM)^d^			TGFβ inhibition by HEK-Blue cells (IC_50_: nM)^e^			Blocking^c^
	LC	HC	TGFb1	TGFb2	TGFb3	TGFb1	TGFb2	TGFb3	
rbt4A11	–	–	> 5000^a^	1.6	1.2	> 667^b^	0.9	0.1	Complete
h4A11.v1	IGKV4-1*01	IGHV3-48*01	> 5000^a^	4.6	14.4	> 667^b^	> 667^b^	> 667^b^	Incomplete
h4A11.v2	IGKV1D-39*01	IGHV3-48*01	> 5000^a^	1.4	13.3	> 667^b^	> 667^b^	> 667^b^	Incomplete
h4A11.v3	IGKV4-1*01	IGHV4-59*06	> 5000^a^	9.6	12.1	> 667^b^	3.9	0.2	Complete
h4A11.v4	IGKV1D-39*01	IGHV4-59*06	> 5000^a^	10	10.1	> 667^b^	1.9	0.1	Complete
h4A11.v5	IGKV3-20*01	IGHV3-48*01	> 5000^a^	12.1	13.9	> 667^b^	> 667^b^	> 667^b^	Incomplete
h4A11.v6	IGKV2-24*01	IGHV3-48*01	> 5000^a^	8.4	12.5	> 667^b^	> 667^b^	> 667^b^	Incomplete
h4A11.v7	IGKV3-20*01	IGHV4-59*06	> 5000^a^	6.3	9.7	> 667^b^	0.9	0.2	Complete
h4A11.v8	IGKV2-24*01	IGHV4-59*06	> 5000^a^	8.2	14.1	> 667^b^	4.8	0.7	Complete

^a^The binding was not observed at the highest TGFβ1 concentration (5 nM); therefore, the reported KD are lower limits.

^b^The maximun inhibition did not achieve over 90% at the highest antibody concentration (667 nM); therefore, the reported IC50 are lower limits.

^c^The complete blocking or incomplete blocking was defined by TGFβ2 and TGFβ3 inhibition greater than 90% or less than 70%, respectively, at the highest antibody concentration.

^d^The affinity constant (K_D_) for each variant is calculated using a global fitting model and reported as a single value for the whole data set with an average fitting standard error of 10%.

^e^The half-maximal inhibitory concentration (IC_50_) for each variant from quadruplicate experiments is calculated using nonlinear regression with a four-parameter variable slope curve fit and reported as a single value with an average fitting standard error of 10%.

### Functional characterization of humanized 4A11 variants

Each humanized 4A11 variant was purified and characterized by measuring its binding affinity and blocking activity against all three mature TGFβ isoforms. To minimize the avidity effect on binding, the affinity of each variant was measured by real-time Biacore SPR using label-free TGFβ in solution with low level of antibody bound to the biosensor surface, as previously described^6^. Further, the blocking activity of the variant IgGs to inhibit mature TGFβ induced TGFβ receptor dependent signaling was determined using reporter cell lines (HEK-Blue™ TGFβ)^1^.

After initial characterization, we observed that all eight primary humanized 4A11 variants preserved the same selectivity profiles as parental antibody (rbt4A11) by binding to human mature TGFβ2 and TGFβ3, but not TGFβ1 (Table 1). Although the humanized variants differed slightly in binding affinity compared to rbt4A11, they retained picomolar binding affinity. However, dramatic differences of blocking activity in the cell-based assay for these humanized variants were observed, which was unexpected because the variants contained almost identical CDRs and closely matched germline frameworks. After detailed analysis, we concluded that humanized variants (v3, 4, 7, and 8) using the heavy chain germline (IGHV4-59*06) as an acceptor framework maintained similar potency compared to rbt4A11, whereas the humanized variants (v1, 2, 5 and 6) using the other heavy chain germline (IGHV3-48*01) significantly lost their blocking activity and also turned into incomplete blockers without reaching maximum inhibition at the highest antibody concentration (< 70%). The usage of different light chain germlines had minimal impact on the blocking activity. Among all eight primary humanized variants, v7 had the most desirable affinity (TGFβ2_K_D_: 6.3 pM; TGFβ3_K_D_: 9.7 pM) with the best blocking activity (TGFβ2_IC_50_: 0.9 nM; TGFβ3_IC_50_: 0.2 nM), and was pursued further as the lead candidate^1^. This result pointed out that the usage of two different yet closely related human heavy chain germlines in the humanization led to the dramatic difference in their blocking activities.

### Crystal structures of TGFβ2 in complex with antigen-binding fragments (Fabs) of h4A11.v2 and h4A11.v7

**Figure 2:**
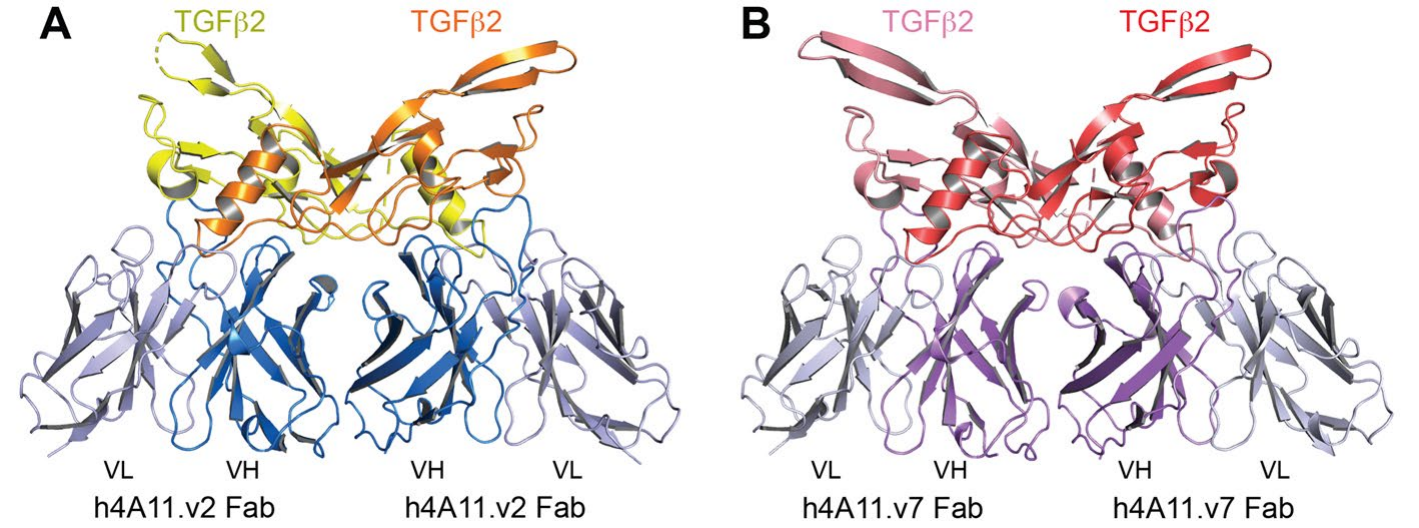
The structures of h4A11.v2 and h4A11.v7 Fabs in complex with TGFβ2 are similar. Overall structures of (**A**) TGFβ2-dimer:h4A11-v2 Fab complex and (**B**) TGFβ2-dimer:h4A11-v7 Fab complex are very similar with RMSD of 1.04 Å for all atoms (only VH-VL regions in the Fab are shown for clarity).

To further investigate how the heavy chain germline acceptor framework differences in the humanized 4A11 variants resulted in significant functional differences in blocking TGFβ2 and TGFβ3, we selected one of the incomplete blockers (h4A11.v2) and the top complete blocker (h4A11.v7) to obtain crystal structures of Fab fragments in complex with TGFβ2. We reported the crystal structure of the h4A11.v7 Fab in complex with TGFβ2 recently^1^. To investigate any structural differences in the TGFβ2:Fab complexes for both v2 and v7 variants, we crystallized and solved the x-ray crystal structure of TGFβ2 in a complex with Fab from h4A11.v2 to 2.9 Å resolution (Table 2). The overall structures of both v7 and v2 complexes are very similar, with two Fab fragments binding to two TGFβ2 molecules and the epitope of each Fab containing contributions from both TGFβ2 monomers in the dimer for the h4A11.v2-Fab, as previously reported for the h4A11.v7-Fab (Fig. 2). The overall RMSD for all atoms in TGFβ2 and the variable regions (Fv) of the complexes is 1.04 Å. The constant regions of the Fabs were excluded from these calculations because the wide variation in elbow angle of Fabs distorts the overall alignments in an irrelevant way^7^.

**Table 2:** Data collection and refinement statistics.

	TGFβ2:h4A11.v2-Fab complex
**Data collection**	
Space group	P 2 21 21
Cell dimensions	
a, b, c (Å)	60.15, 103.48, 196.25
a, b, g (°)	90, 90, 90
Resolution (Å)	45.95–2.90 (3.08–2.90)*
R_pim_	0.021 (0.389)
I/σI	11.6 (1.34)
Completeness (%)	98.50 (99.70)
Redundancy	4.0 (4.3)
CC_1/2_	0.998 (0.663)
**Refinement**	
Resolution (Å)	2.90
No. reflections	27,582 (2630)
R_work_/R_free_	0.2080 (0.3217)/0.2860 (0.3482)
No. atoms	8200
Protein	8152
Ligand/ion	0
Water	48
*Average B-factors*	77.40
Protein	77.40
Ligand/ion	0.00
Water	64.90
**R.m.s. deviations**	
Bond lengths (Å)	0.013
Bond angles (°)	1.229

1 crystal used the dataset. *Values in parentheses are for highest-resolution shell.

**Figure 3:**
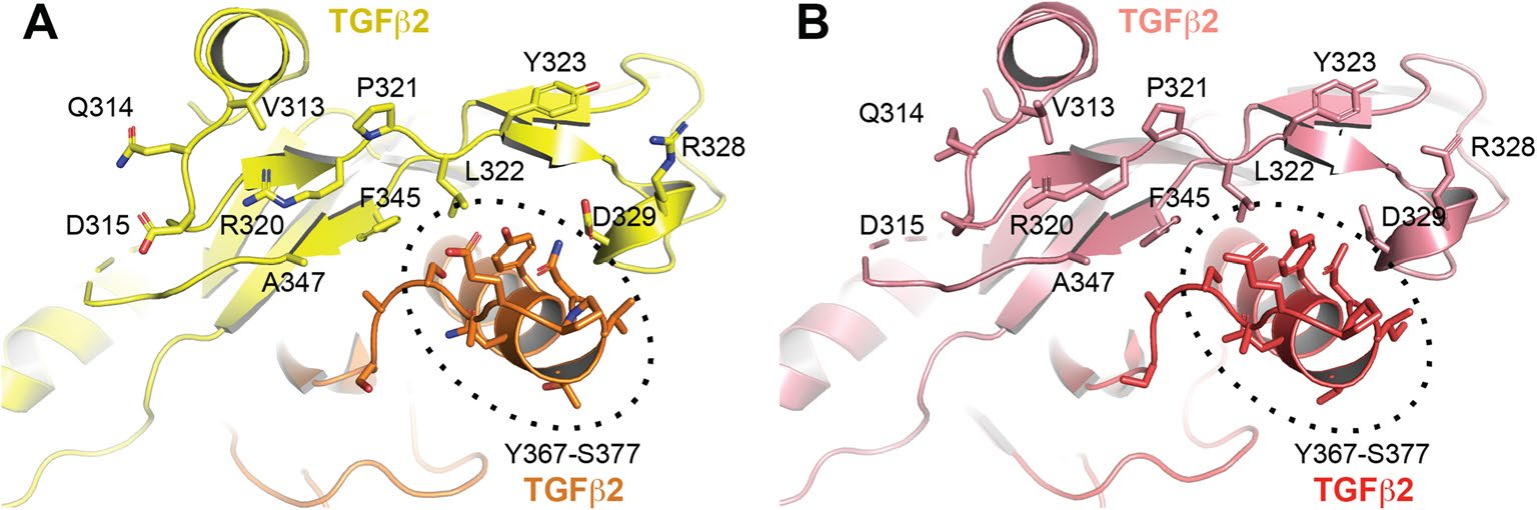
The epitopes of h4A11.v2 and h4A11.v7 on TGFβ2 are identical. The residues in TGFb2 dimer that form the epitopes of (**A**) h4A11.v2 and (**B**) h4A11.v7 are highlighted as sticks and labelled.

**Figure 4:**
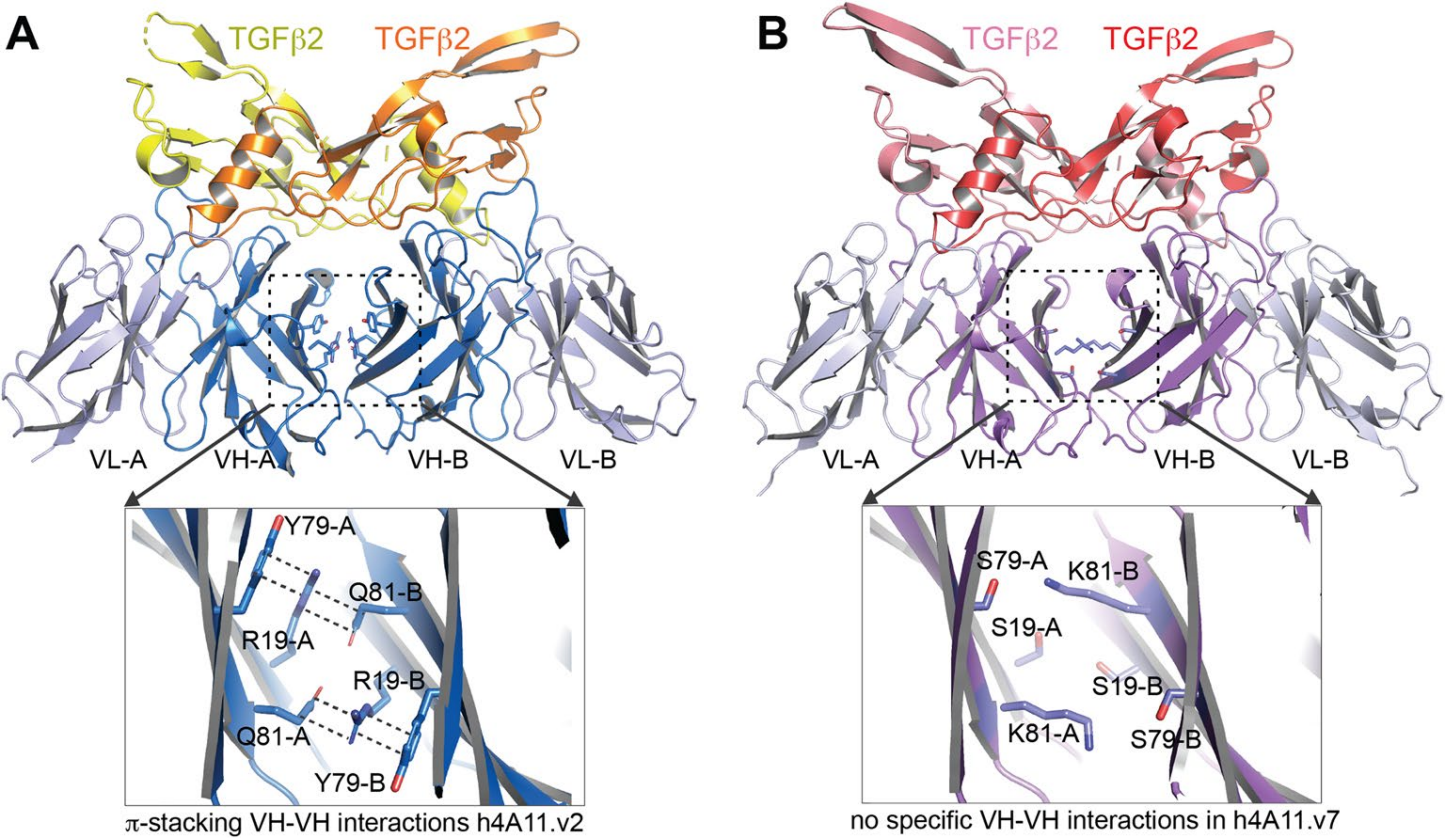
Molecular interactions between h4A11.v2 and h4A11.v7 Fabs bound to TGFβ2. (**A**) π-stacking between Y79 and R19 from one heavy chain A and Q81 from the second heavy chain B and vice versa results in a stable interaction between the two heavy chains of the Fabs in the h4A11.v2:TGFβ2 complex. (**B**) Aforementioned π-stacking is disrupted in h4A11.v7:TGFβ2 complex because of the substitution in the framework region of the heavy chain residues to S79, S19 and K81.

An analysis of TGFβ2:h4A11.v2-Fab and TGFβ2:h4A11.v7-Fab crystal structures revealed that the epitopes of both h4A11.v2 and h4A11.v7 antibodies on the TGFβ2 dimer are identical (Fig. 3). This, along with identical CDRs between the two variants, suggested that the functional differences observed between the two variants cannot be explained by a potentially altered epitope. Further analysis of the two structures revealed an intriguing interaction of the 2 heavy chain molecules in the TGFβ2:h4A11.v2-Fab complex with each other, which was not present in the TGFβ2:h4A11.v7-Fab complex. In the TGFβ2:h4A11.v2-Fab complex structure, Y79, R19 from one heavy chain molecule form a ternary π-stacking interaction with Q81 from the other heavy chain molecule and vice versa, resulting in a pseudo-symmetric interaction (Fig. 4A), which likely orients the relative positions of the two heavy chains and the conformation of the Fabs relative to TGFβ2 and alters the dynamics of the h4A11.v2 Fab when bound to TGFβ2 in solution, compared to the h4A11.v7 Fab. Supporting this hypothesis, in the TGFβ2:h4A11.v7-Fab complex structure, the corresponding residues in the heavy chain framework region are S19, S79 and K81, which do not allow for the specific interactions observed in the TGFβ2:h4A11.v2-Fab complex (Fig. 4B). Although the distances between the Cα atoms of the respective residues at each position (19–19, 79–79, 81–81) in the heavy chains of both the h4A11.v2 and h4A11.v7 Fabs are almost identical in the two structures, we hypothesized that in solution, the interactions between the side chains at 19, 79 and 81 among the 2 heavy chain molecules and subtle effects on the conformations of the Fabs may be the difference between a functional ‘complete blocker’ and an ‘incomplete blocker’, where the slight differences induced in the dynamics of the TGFβ2 dimer itself due to the presence or absence of the subtle Fab–Fab interaction could play a role. For such an interaction, we assume that two Fabs from the same antibody molecule would bind to a TGFβ2 dimer in solution since the rbt4A11 antibodies have monovalent interactions with dimeric TGFβ2 and TGFβ3. To test this hypothesis, we performed step by step mutagenesis to try and convert a v2-like function (incomplete blocking) to a v7-like function (complete blocking) with an increasing number of mutations listed in Table 3. We hypothesized that a simple R19S substitution in h4A11.v2 would disrupt the π-stacking interaction, and so would Q81N or Q81A. In addition, to convert a v7-like function to a v2-like function, we hypothesized that we would need to introduce a greater number of mutations at positions 19, 79, 81 to re-build the stabilizing interactions of a v2-like molecule. In this process, we also included an aromatic sidechain W at position 79, as it could result in a π-stacking interaction similar to Y in the TGFβ2:h4A11.v2-Fab structure (Fig. 4).

**Table 3:** Functional characterization of humanized 4A11 version 2 and version 7 heavy chain framework mutation variants.

Variants	Mutation	TGFβ binding by Biacore SPR (K_D_: pM)^d^			TGFβ inhibition by HEK-Blue cells (IC_50_: nM)^e^		Blocking^c^
		TGFb1	TGFb2	TGFb3	TGFb2	TGFb3	
h4A11.v2.1	R19S	> 5000^a^	2.2	0.6	0.7	0.1	Complete
h4A11.v2.2	Q81N	> 5000^a^	4.5	6.0	2.2	0.4	Complete
h4A11.v2.3	Q81A	> 5000^a^	3.4	5.5	1.7	0.7	Complete
h4A11.v2.4	R19S, Y79S	> 5000^a^	1.4	0.6	2.2	0.2	Complete
h4A11.v2.5	R19S, Q81S	> 5000^a^	1.7	0.6	0.7	0.1	Complete
h4A11.v2.6	R19S, Y79S, Q81S	> 5000^a^	1.9	0.5	1.7	0.3	Complete
h4A11.v7.1	S19R, S79Y	> 5000^a^	10.7	40.5	> 667^b^	> 667^b^	Incomplete
h4A11.v7.2	S19R, S79W	> 5000^a^	9.6	22.8	> 667^b^	> 667^b^	Incomplete
h4A11.v7.3	S19R, S79Y, K81Q	> 5000^a^	5.7	8.0	> 667^b^	> 667^b^	Incomplete
h4A11.v7.4	S19R, S79Y, K81R	> 5000^a^	11.2	18.8	> 667^b^	> 667^b^	Incomplete
h4A11.v7.5	S19R, S79W, K81Q	> 5000^a^	5.6	7.2	> 667^b^	> 667^b^	Incomplete
h4A11.v7.6	S19R, S79W, K81R	> 5000^a^	9.7	15.6	> 667^b^	> 667^b^	Incomplete

^a^The binding was not observed at the highest TGFβ1 concentration (5 nM); therefore, the reported KD are lower limits.

^b^The maximun inhibition did not achieve over 90% at the highest antibody concentration (667 nM); therefore, the reported IC50 are lower limits.

^c^The complete blocking or incomplete blocking was defined by TGFβ2 and TGFβ3 inhibition greater than 90% or less than 70%, respectively, at the highest antibody concentration.

^d^The affinity constant (K_D_) for each variant is calculated using a global fitting model and reported as a single value for the whole data set with an average fitting standard error of 10%.

^e^The half-maximal inhibitory concentration (IC_50_) for each variant from quadruplicate experiments is calculated using nonlinear regression with a four-parameter variable slope curve fit and reported as a single value with an average fitting standard error of 10%.

### Generation and functional characterization of structure-guided h4A11.v2 and h4A11.v7 heavy chain framework variants

**Figure 5:**
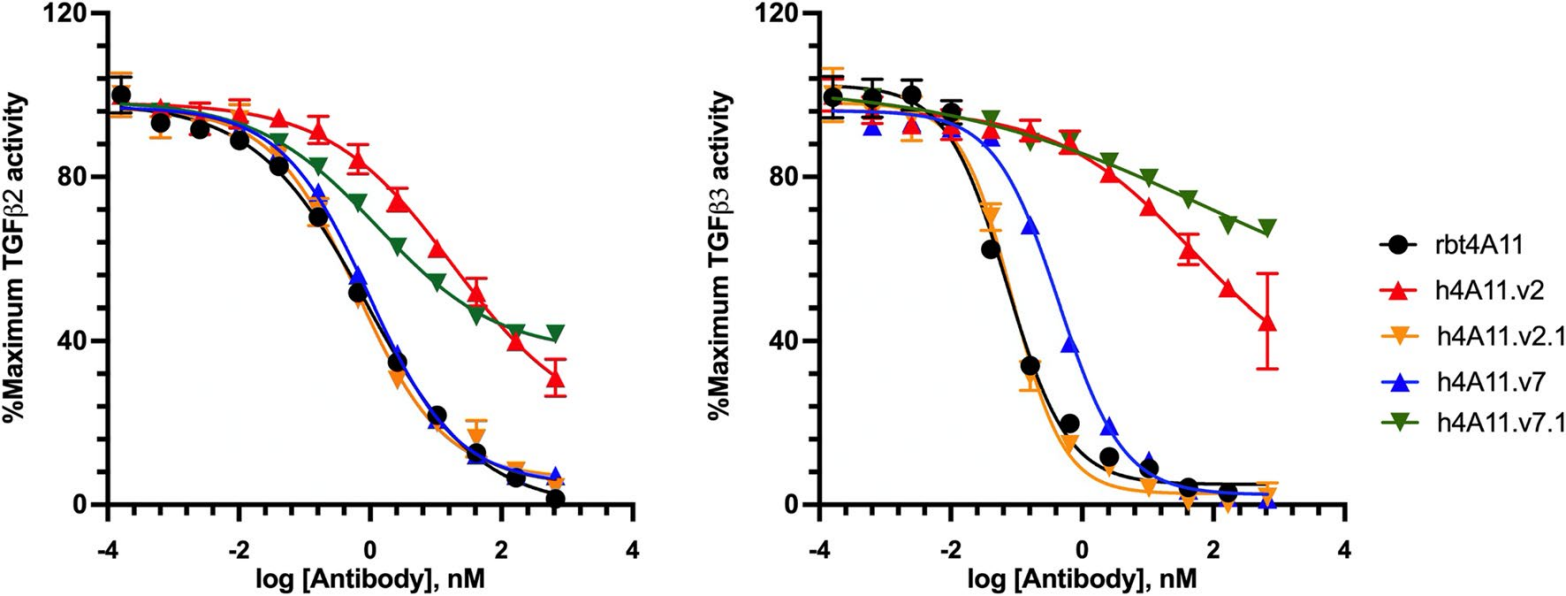
Dose-dependent inhibition of TGFβ by humanized 4A11 variants in HEK Blue™ TGFβ reporter cell-based assay. Human mature TGFβ2 (left) or TGFβ3 (right) isoform was incubated with a serial dilution of humanized 4A11 variants in quadruplicate to generate a dose-dependent inhibition curve. The response was normalized to maximum TGFβ activity (%) and mean activity ± standard deviation was plotted as a function of antibody concentration. The variants exhibiting less than 10% of maximum TGFβ activity at the highest antibody concentration were complete blocker (e.g., rbt4A11, h4A11.v2.1, h4A11.v7), whereas those reaching greater than 30% of maximum TGFβ activity were incomplete blockers (e.g., h4A11.v2, h4A11.v7.1).

To test the hypothesis in accordance with our structural analysis, a new panel of variants was generated with single or multiple mutations at position 19 (HC-FWR1), 79 and 81 (HC-FWR3) of h4A11.v2 and h4A11.v7. Following expression and purification, all variants were fully characterized and compared with parental rbt4A11, h4A11.v2 and h4A11.v7. As predicted from our structural analysis, all h4A11.v2-based variants (h4A11.v2.1–h4A11.v2.6) demonstrated binding affinity improvement as well as regained complete blocking activity in the cell-based assay against TGFβ2 and TGFβ3 (Table 3). All three single mutation variants (v2.1_R19S; v2.2_Q81N; v2.3_Q81A) were sufficient to restore complete blocking function similar to rbt4A11 (v2.1 in Fig. 5). Furthermore, by adding other mutations to R19S (v2.4_ R19S, Y79S; v2.5_ R19S, Q81S; v2.6_ R19S, Y79S, Q81S), did not further change the effect of the individual mutations, suggesting the critical role of positions 19, 79 and 81 in the observed h4A11.v2 heavy-chain interactions upon TGFβ2 and TGFβ3 binding. In contrast, all h4A11.v7-based variants (h4A11.v7.1–h4A11.v7.6) with double mutations (v7.1_S19R, S79Y; v7.2_S19R, S79W) and triple mutations (v7.3_S19R, S79Y, K81Q; v7.4_S19R, S79Y, K81R; v7.5_S19R, S79W, K81Q; v7.6_S19R, S79W, K81R) appeared to have a broad range of affinity drop (4–34 fold) compared to rbt4A11 and turned into incomplete blockers against TGFβ2 and TGFβ3 (v7.1 in Fig. 5). These results supported the important functional role of short sidechain amino acids at human framework positions 19 and 79 in the active h4A11.v7 molecule.

## Discussion

In our current work, we identified a unique and interesting phenomenon during humanization of an allosteric antibody against TGFβ2 and TGFβ3, where a single amino acid in the framework region regulates antibody function. This work highlights how subtle differences in framework regions with no effect on antigen binding can dramatically alter antibody function in allosteric ligand blocking. We also demonstrate that it is possible to convert the inhibitory properties of the humanized antibodies by a single amino acid change in framework.

Functional antibodies impart their effects on their targets primarily through the interaction of their CDRs with the antigen. For symmetric, multimeric antigens, where appropriate relative orientations of each monomer are important for binding to partner proteins and for signaling, the existence of multiple epitopes of the antibody (Fab) and the multimeric nature of the interaction between antigens and Fabs could have consequences for signaling. This may offer clues to further design favorable properties for antibodies by varying the regions of antibodies beyond CDRs. As illustrated in this report, the interactions (or lack thereof) between the heavy chain framework regions in two Fabs can have a profound impact on signaling.

Although humanization is a well-established art in the field of antibody engineering, potential learnings especially from allosteric antibodies against antigens that function as multimers could be useful in improved antibody design. In this work, we have introduced and demonstrated an effective approach to humanize the rabbit monoclonal antibody 4A11 using the combinations of CDR grafts and framework shuffling. This approach has been successful in our humanization of multiple rabbit mAbs (data not shown). During the humanization process, we usually select the variants with activities similar to parental antibodies to move forward. However, in the humanization of 4A11, the variants displayed intriguing properties with similar binding affinity, but dramatically different blocking activity in cell based activity assays. The crystal structures of h4A11.v2 and h4A11.v7 Fabs bound to TGFβ2 have further delineated the mechanism of the allosteric inhibition that is dependent on homotypic interactions between framework regions of heavy chains. The subtle difference in the human framework impacts the interactions between the heavy chains framework regions of the two Fabs of humanized 4A11 that leads to the loss of activity in h4A11.v2, but not h4A11.v7.

Interaction between framework regions of the heavy chain of Fabs within a functional antibody has been observed before, for example, the anti-HIV-gp120 neutralizing Fab, 2G12 which was isolated from a patient that was resistant to several strains of HIV^8^. In the case of 2G12, the variable domain of one heavy chain undergoes a domain exchange with the variable domain of the 2nd light chain in the antibody and vice versa resulting in a dimerized Fab^9^. In addition, in 2G12, the typically surface exposed framework regions of the heavy chain are also extensively modified to form a specific stable interaction between the framework regions of the two heavy chains. This places the paratopes of the antibody in a defined, rigid geometry, that allows specific recognition of the carbohydrate clusters on the HIV gp120, which would not be possible with a typical IgG scaffold, where there are no stabilizing interactions between the heavy chain variable domains.

Our work, in addition to the 2G12 example above, suggests that the framework regions offer yet another region in an antibody for engineering interactions between Fabs and modulating antibody function. This could especially be useful where antigens are multimeric and restricting the paratopes on Fabs in a defined orientation to match a geometrically restricted and repeated epitope in a multimeric target could be useful to impart agonistic or antagonistic properties to the antibody, on top of binding properties imparted by CDRs, based on the molecular mechanism of signaling of the antigen and its effector molecules.

## Methods

### Humanization, IgG variants cloning, expression and purification

The variable regions of rabbit antibody 4A11 (rbt4A11) amino acid sequence were aligned to its closest human germlines using informatics tool (AbGrafter, Genentech) which covered all available immunoglobulin IGKV and IGHV mammalian human germline genes (*Homo sapiens*) in the international immunogenetics information system^10^. The closest human IGKV and IGHV germline frameworks identified through the process were then served as the acceptor frameworks for grafting rbt4A11 light chain and heavy chain hypervariable regions with the corresponding rabbit framework residues at the Vernier zone respectively^3^. By mixing and matching these grafting acceptor frameworks, a total combination of eight humanized 4A11 variants (h4A11.v1–h4A11.v8) were generated.

The coding sequences of each humanized variant’s variable region were generated by DNA synthesis and cloned into human IgG1 expression vectors (Genewiz). Small-scale IgG expressions and purifications were done in a high-throughput format. Briefly, IgG expressions were transiently conducted in Expi293F cells (Thermal Fisher Scientific), followed by a 2-step purification using Protein A affinity chromatography (MabSelect SuRe™, Cytiva) and analytical size exclusion chromatography (SEC, Cytiva). Quality control of antibody’s purity was determined by SDS-PAGE with Coomassie blue staining, A280 absorbance to measure protein concentration and aggregation analysis by SEC, as previously described^11,12^.

### Expression, purification and crystallization of human TGFβ2 and h4A11.v2 /h4A11.v7 Fab

The mature form of human TGFβ2 was produced as previously reported^13^. LAP-TGFβ2 was expressed with a CHO TI stable cell line. Protein was purified with Ni–NTA, then was acidified and dialyzed in 0.1 M sodium citrate, pH 3 and loaded onto a 5 ml SP HP column. The fractions that contained the protein of interest from the SP pool were then further purified with Vydac C4 RP-HPLC. The fraction containing mature TGFβ2 was dialyzed directly into 1 mM HCl pH 3.0.

The Fab fragments of anti-TGFβ2 (h4A11.v2 and h4A11.v7) were expressed in *E. coli* overnight at 30ºC under the control of the PhoA promoter. The cells were pelleted by centrifugation at 6000 rpm for 15 min and lysed by microfluidization in PBS supplemented with 25 mM EDTA and 1 mM PMSF. Cell debris was removed by centrifugation at 10,000 rpm for 1 h at 4 °C. The resulting supernatant was run through a Protein G column equilibrated in PBS, and eluted with 0.58% acetic acid. Protein fractions were further purified by ion exchange chromatography (SP-sepharose) in 20 mM MES (pH 5.5) and eluted with a gradient from 0 to 250 mM NaCl.

The TGFβ2 and h4A11.v2 Fab complex was mixed at 1:1 molar ratio and further purified using a Superdex-200 column equilibrated in 25 mM Tris–HCl (pH 7.5) and 200 mM NaCl. For crystallization, the complex samples were concentrated to 10 mg/ml. The h4A11.v2 Fab/TGFβ2 crystals were obtained by vapor diffusion at 19 °C with mixing equal volumes of protein plus 15% PEG 4000 and 0.1 M Na Cacodylate pH 6.0 well solution. Crystals were cryoprotected with 25% glycerol. Data set was collected at the Advanced Light Source beamline 5.0.2 and processed with the HKL package^14^. Crystal structures were solved by molecular replacement using Phaser^15^. The refined coordinates of the TGFβ2 structure PDB file (6I9J) served as the search probe for the structure for TGFβ2^16^. Subsequently, the constant (Fc) and variable (Fv) portions of the Fab were placed separately using Phaser and underwent initial rounds of rigid-body refinement with Phenix^17^. The model went through several iterative rounds of adjustment with Coot^18^. Atomic models were then built and refined with Phenix^17^.

### TGFβ binding affinity measurement

The binding affinity of each humanized 4A11 (h4A11) variant was determined by surface plasmon resonance (SPR) technology (Biacore™-T200, Cytiva). Briefly, Series S sensor chip Protein A (Cytiva) was utilized to capture each version of h4A11 antibody on a different flow cell (FC) to achieve approximately 90 response units (RU), followed by the injection of five-fold serial dilutions of each human mature TGFβ isoform (PeproTech; 0.008 nM to 5 nM) in HBS-EP buffer (100 mM 4-(2-hydroxyethyl)-1-piperazineethanesulfonic acid (HEPES) pH 7.4, 150 mM NaCl, 3 mM EDTA, 0.05% (v/v) Surfactant P20) with a flow rate of 100 μl/min at 37 °C to reach a relative low maximum binding response (Rmax) around 30 RU. The association was monitored by a standard 3 min observation, whereas the dissociation was extended to a much longer time (60 min) to receive sufficient decay for accurate off-rate measurement. The sensorgrams were recorded, processed by reference and blank subtraction, and evaluated by a simple one-to-one Langmuir binding model (Biacore T200 evaluation software version 2.0) to determine the equilibrium dissociation constant (K_D_).

### TGFβ inhibition cell-based assay

The blocking potency of each h4A11 variant against each human mature TGFβ isoform was evaluated by a HEK-Blue™ TGFβ cell-based reporter assay. HEK-Blue™ TGFβ cells (InvivoGen) were generated by stable transfection of HEK293 cells with the human TGFβ receptor 1, Smad3, and Smad4 genes, and further expressed a Smad3/4-binding elements (SBE)-inducible secreted embryonic alkaline phosphatase (SEAP) reporter gene. Before experiments, cells were maintained in DMEM high glucose medium containing 10% fetal bovine serum (VWR), 2 mM L-glutamine, 50 units/mL penicillin, 50 μg/mL streptomycin, 100 μg/ml Normocin™, 30 µg/ml of blasticidin, 200 µg/ml of Hygromycin B Gold and 100 µg/ml of Zeocin™ (InvivoGen) at 37 °C ± 0.5 °C with 5% CO_2_. On the assay day, cells were seeded in cells were seeded in Greiner Bio-One™ CellStar™ 384-Well, cell culture-treated, flat-bottom microplates at a density of 9000 cells per well in the test medium (DMEM high glucose supplemented with 10% heat inactivated fetal bovine serum, 50 units /mL penicillin, 50 μg/mL streptomycin, and 2 mM glutamine) and incubated for 15 min at the condition described above. Each antibody variant, fourfold serially diluted for 12 points (final 0.000159 nM to 667 nM), was incubated with each human mature TGFβ isoform (PeproTech; final 20 pM) for 1 h, followed by transferring to the plates containing cells. The SEAP in the supernatant was measured with Quanti-Blue (InvivoGen) per manufacturer’s instructions after 37 °C incubation for 18–22 h. All experiments were done in quadruplicates, and the maximum or minimum response was determined by TGFβ or test media only respectively for normalization. The half maximal inhibitory concentration (IC_50_) values were then calculated by fitting the titration curves ([Antibody variant] vs. % Maximum TGFβ activity) using a Prism variable slope four parameters model (GraphPad Software).
